# Industrial metabolism of iron and carbon with substance flow analysis in iron and steel industry

**DOI:** 10.1371/journal.pone.0327904

**Published:** 2025-08-07

**Authors:** Junwen Chen, Qing Sun

**Affiliations:** 1 Hubei Key Laboratory of Green Intelligent Manufacturing Technologies of Battery Key Materials, Hubei University of Automotive Technology, Shiyan, PR China; 2 Key Laboratory of Automotive Power Train and Electronics, Hubei University of Automotive Technology, Shiyan, PR China; Khalifa University, UNITED ARAB EMIRATES

## Abstract

Iron and carbon are inextricably linked from the source to the destination in iron and steel system. With the shortage of iron ore and severe pollution caused by emissions, it is pressing to improve resource efficiency and reduce pollution. To comprehensively evaluate resource efficiency and understand the metabolic processes of iron and carbon, SFA (Substance flow analysis) was considered as an effective assessment method to assess the resource efficiency. In this paper, a widespread SFA model is proposed, and four evaluation indicators are proposed to appraise the metabolic processes of iron and carbon. According to the data of a state-owned and large-scaled steel enterprise in China, metabolic network of iron and carbon are established. The results of this study showed that the resource utilization efficiency of iron is 86.77%, and that of carbon is 0.33%. Focus is given onto the metabolic processes of iron and carbon in BOF and EAF, as well as the iron-carbon metabolism nexus. When considering the material and energy metabolism of the upstream process, the molten iron ratio is inversely proportional to the electricity consumption intensity and directly proportional to the carbon emission intensity. The average emission intensity will decrease 83.00 kg CO_2_/t molten steel if the molten iron ratio decreases 0.05 in EAF, while electricity consumption intensity increases 32.00 kwh/t molten steel.

## 1. Introduction

China’s crude steel production with 1064.80 Mt ranked first in the world in 2020, which is approximately 100 times that of India, according to the steel statistical yearbook provided by World Steel Association [1]. The routes for crude steel production can be divided into two types: BF-BOF and EAF. The BF-BOF route needs to consume approximately 1.37 t iron ore, 0.78 t coal, 0.27 t limestone, and 0.13 t scrap steel for producing 1.00 t of crude steel [2], and discharge 2.10 t carbon dioxide [3]. The average consumption can reach 0.59 t scrap steel/crude steel, 0.15 tons coal/crude steel, 88.00 kg limestone/crude steel, and 2.30 GJ electricity/crude steel in EAF route. Due to the differences in technology and equipment, CO_2_ emissions intensity is 2148.00 kg CO_2_/t crude steel in China, 1708.00 kg CO_2_/t crude steel in Germany, 1080.00 kg CO_2_/t crude steel in Mexico, and 1736.00 kg CO_2_/t crude steel in the the United States [4]. However, ISI must face huge energy consumption and serious environmental pollution due to massive exploitation and smelting of iron ores, which is a major industry for greenhouse gas emissions [5,6]. Hence, estimating and improving the resource utilization efficiency of iron and carbon is of great significance for sustainable development [7].

The SFA method has been considered as an effective method to analyze the resource utilization efficiency of material and energy for guiding industrial policies and economic activities in a specific system. It is typically used in conjunction with IO, LCA, environmental assessment indicators, parameter [8,9], design [10] or optimization [11,12]. What’s more, at the city or country level, SFA can be used to help understand the metabolic processes of urban resources, energy and the connection between various sections of metabolism. Subjects may be metallic and nonmetallic elements, such as iron and steel, lithium, sulfur, carbon, etc. Steel flow and stock in all provinces of China can be analyzed by the SFA method [13], however, large range cannot reflect the process of material metabolism in industrial parks or factories. As a result, at present, most studies of material and energy metabolism are more favored by researchers at the eco-industrial park or factory level.

Some scientific and appropriate indicators have been developed based on different ranges of metabolic models to assess environmental impacts and make corresponding improvements, mainly focusing on the nexus between substances metabolism in industrial systems [14,15]. Dai et al applied SFA model to evaluate the structure, resource efficiency of ISI, and pointed out that the excessive consumption of primary energy has led to serious environmental degradation [16]. Zhong et al used SFA model to build a complex network and analyzed the evolution of features and structure of international iron flow [17]. Zhang et al adopted industrial symbiosis technology with SFA method to reduce carbon emissions [18]. Ma et al founded the stocks and flows model with SFA to analyze carbon flows [19]. Park et al established a dynamic SFA of steel and discussed the impact of product life and recycling rates on scrap demand [20]. Li et al calculated the iron stock based on dynamic SFA to provide data on the recycling of secondary iron resources [21]. These studies provide valuable references for the SFA of iron and carbon, nevertheless, but only single element is considered. Iron and carbon are the most important elements in iron and steel system. It is necessary to analyze their metabolic process and relationship for energy conservation and emission reduction.

Although there are few joint research studies that consider two elements simultaneously, they can reflect their interactions. Gao et al adopted SFA model to quantify relations among metabolic processes for analyzing the environmental load of elements nitrogen, phosphorus and sulfur [22]. Liu et al studied the evolution of lithium and cobalt metabolism with dynamic material flow in lithium-ion batteries system [23]. Wu et al evaluated the chromium and carbon metabolic behavior with ecological network analysis [24]. Iron and carbon are the most important elements in iron and steel system, while the interactions are unclear and need to be studied in depth. The BF-BOF route and the EAF route are quite different in metabolic behaviours of material and energy. Hot metal is widely used in EAF due to huge electricity consumption and shortage of scrap steel. The EAF needs to be studied on the metabolic behaviours of material and energy. Hence, studying and comparing the iron-carbon metabolism of these two steelmaking methods will be an important guideline for the development of iron and steel enterprises in the future. In summary, SFA method has been considered as an effectual and comprehensive assessment method for the material, energy and emission flow network in the ISI. To further understand the metabolic processes of iron and carbon, we have done the following work. The iron flow and carbon emission flow are constructed according to the defined boundary of the steel manufacturing system, and the main inputs and outputs of each process are marked in the flow network.

The remainder of this paper is organized as follows. Section 2 introduces the methodology including boundary of this case, general model for SFA and process of assessment and indexes. Section 3 presents the data source of the case. Section 4 presents the results and discussion. Finally, Section 5 provides the conclusions and contributions of this study.

## 2. Methodology

### 2.1 Scope and boundary

**Fig 1:**
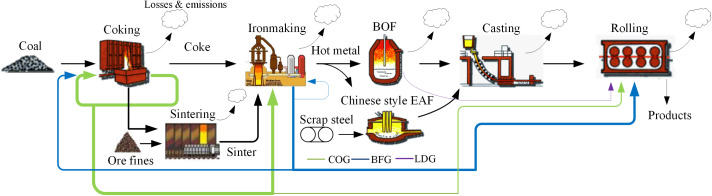
Scope and boundary of iron and steel system.

The ISI is a representative high-temperature production process with high energy consumption and high pollution. The main reactions involved in each stage are diverse in all process, and the smelting stage is dominated by chemical reactions and the rolling stage is dominated by physical changes. All processes must be efficaciously connected with the information flow, material flow and energy flow, which has the characteristics of complexity, mutation and uncertainty. As shown in Fig 1, a typical iron and steel plant mainly includes the following processes: (1) preparation system including sintering, pelleting and coking; (2) ironmaking; (3) steelmaking including BOF and EAF; (4) casting. (5) rolling including hot rolling and cold rolling. Each process can be marked with the input and output of materials and energy, losses, emission, flow direction.

### 2.2 General model for SFA

**Fig 2:**
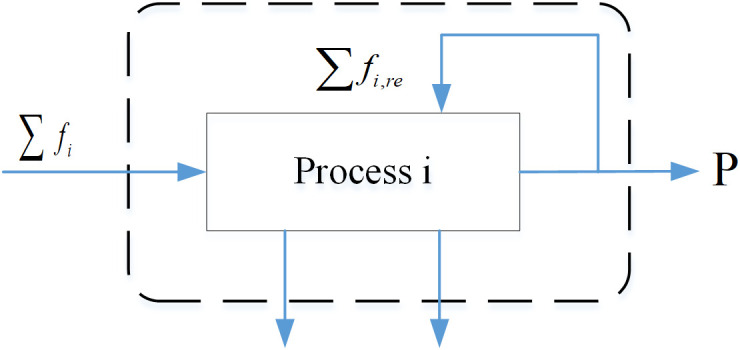
General model for SFA.

SFA focuses on quantifying material input and output in the socioeconomic or environmental system and investigating the major pathways and associated processes, including material storage, fluxion and ultimate flow in the environment. The final objective is to maintain the sustainability of industrial metabolism. By establishing material flow accounts, it is possible to optimize the management of elements, raw materials, products and even waste for maximize resource utilization and minimize environmental impact from mining, production, consumption, recycling to end-of-life. The general model for SFA including several processes is displayed in Fig 2, and each process has its input and output, wastes and recycling flows. Take process i as an example: (1) $M_{i}$ indicates the input from the external environment to process i, such as coke, sinter, pellet, etc. in the ironmaking process; (2) $M_{i,r}$ represents the recycled substance, such as the scale skin and muddy dust; (3) $M_{W,i}$ implies waste substances, such as furnace dust, sludge, dust, etc; (4) $M_{B,i}$ illustrates byproduct substances, such as BFG, LDG and slag; (5) $M_{P}$ denotes the final product, such as hot metal or molten iron.

### 2.3 Process of assessment and indexes

**Fig 3:**
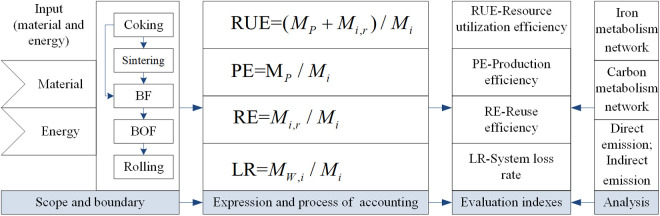
Process of assessment and index.

Through the establishment of evaluation indicators, it is effective to study and understand the state of material flow system, so as to formulate reasonable measures to make full use of resources. Some evaluation indicators for SFA provided effective references for this study. Simultaneously, in order to reasonably and scientifically evaluate the iron and carbon metabolism of the ISI, the following evaluation indicators are formulated, as shown in Fig 3. These assessment metrics include the following: (1) RUE represents resource utilization efficiency of one substance; (2) PE represents production efficiency of one substance; (3) RE means the ratio of recyclable material; (4) LR implies system loss rate.

### 2.4 Iron and carbon accounting

#### 2.4.1 Iron accounting.

The ore and iron-containing materials are the source of iron in the system. The input of iron is calculated according to the grade of ore, as shown in Eq. (1).


$$\sum iron_{in}=\sum \left(ore_{i}\times G_{i}\right)$$ (1)

Where $ore_{i}$ indicate the i-th ore or iron-containing materials, $G_{i}$ indicate the grade of i-th ore or or iron-containing materials. According to the results of iron metabolism, it can be divided into four types: (1) iron in products; (2) iron in recycled materials; (3) iron in waste and (4) system loss. The iron accounting is calculated according to the law conservation of mass, as shown in Eq. (2).


$$\sum Iron_{in}=\sum Iron_{out}^{product}+\sum Iron_{out}^{recycling}+\sum Iron_{out}^{waste}+\sum Iron_{out}^{loss}$$ (2)

#### 2.4.2 Carbon accounting.

Fossil fuels and ores are the main sources of iron and steel systems, and air pollution comes from their consumption. In earlier studies, the IPCC method is used to calculate carbon emissions, only considering carbon emissions from fossil fuel combustion but ignores hidden carbon emissions. Hence, in order to calculate carbon emissions more accurately, the law conservation of mass is used to calculate carbon emissions. Carbon inputs end up either in products, or in by-products, or in waste, or in the carbon dioxide.


$$\sum C_{out}=\sum C_{direct}^{CO_{2}}+\sum C_{indirect}-\sum C_{deduction}$$ (3)

Where $\sum C_{out}$,$C_{out}^{CO_{2}}$,$C_{indirect}$ indicate the net $CO_{2}$ emissions, the direct emissions in the form of $CO_{2}$, the input of indirect carbon flow, respectively. $C_{deduction}$ indicates the carbon deduction by products or byproducts utilization. The capital letter C refers to the amount of carbon.

The direct emissions in the form of $CO_{2}$ can be calculated according to Eq.(4):


$$\sum C_{direct}^{CO_{2}}=\sum C_{direct}\times E_{P}\times 44/12$$ (4)

Where $E_{P}$ indicates the equivalence carbon dioxide emission factor, as shown in Table 1.

**Table 1:** Carbon emission factors in this study [25].

Item	Value	Item	Value	Item	Value
Washed coal kg CO_2_/kg	2.69	COG kg CO_2_/GJ	44.40	Diesel kg CO_2_/kg	2.72
Hard coal kg CO_2_/kg	3.09	BFG kg CO_2_/GJ	260.00	Electricity kg CO_2_/kwh	0.85
Coke kg CO_2_/kg	3.14	LDG kg CO_2_/GJ	100.00	–	–

The input of indirect carbon can be calculated according to Eq. (5):


$$\sum C_{indirect}=\sum P_{in}\times E_{P}\times 44/12$$ (5)

Where $P_{in}$ indicates the input flow by byproducts, other materials or energy, etc.

The carbon deduction by products or byproducts utilization can be calculated according to Eq. (6):


$$\sum C_{deduction}=\sum P_{out}\times E_{P}\times 44/12$$ (6)

Where $P_{out}$ indicates the output flow by products, or byproducts, or waste, etc.

## 3. Data source

The data comes from a state-owned and large-scaled steel enterprise in China. The whole ISI includes coking plant, sintering plant, pelleting plant, ironmaking plant, steelmaking plant, continuous casting plant, rolling plant. etc. This enterprise is the largest H-beam production base with the most complete specifications, and the largest gear steel production base, and the largest powder metallurgy production base with the highest added value.

The material preparation system contains the following: three 105 m^2^ sintering machines and two 265 m^2^ sintering machines in sintering area; two 8 m^2^ shaft furnace in pelleting; two 750 m^3^ blast furnaces, four 1080 m^3^ blast furnaces and one 3200 m^3^ blast furnace in ironmaking area; three 50t BOFs, one 60t BOF, two 80t BOFs, three 105 m^2^ sintering machines and two 265 m^2^ sintering machines in sintering area 120t BOFs, one 50t EAF and two 30t EAFs in steelmaking area; ten continuous casting machines and twelve rolling mill groups. The output of crude steel of the enterprise is 8.40 Mt per year. The iron and steel products of plate steel, bar steel, section steel, special steel are 2.81, 2.09, 2.12 and 1.00 Mt, respectively. Statistical data of annual production and material and energy consumptionof all processes are shown in Table 2. Iron and carbon metabolism will be evaluated based on the data and evaluation model to understand the metabolic mechanisms and develop reasonable measures for achieving the goal of maximize resource efficiency and minimize environmental impact.

**Table 2:** Actual statistical data of steel enterprises.

Category	Value	Category	Value	Category	Value
Crude steel (Mt)	8.4	Sinter (Mt)	10.28	Water consumption per ton of steel (t/t)	3.43
Products (Mt)	8.02	Pellets (Mt)	2.35	Power consumption per ton of steel (kWh/t)	405.56
Coke (Mt)	3.37	Lump ore (Mt)	0.92	Gas consumption per ton of steel (GJ/t)	9.66
Scrap steel (Mt)	0.86	Titanium ore (Mt)	0.04	All energy consumption ($\times 10^{9}$kgce)	5.56

## 4. Results and discussion

### 4.1 Comparative analysis of other methods

Table 3 shows the comparison between this paper and other literature in terms of research methods, innovation features, evaluation indicators, and main results. The flow analysis based on SFA is a common analysis method. Carbon emission, energy intensity and energy efficiency are the focus, and the multi-objective optimization with carbon emission as an important index provides valuable guidance for energy conservation and emission reduction in the ISI. However, in this paper, iron metabolic flow and carbon metabolic flow and the relationship between the two are analyzed deeply and carefully. This makes the metabolic flow of the ISI more clear and understandable, so that potential energy saving and emission reduction pathways can be found.

**Table 3:** Comparison with other methods.

Methods	Characteristic	Evaluation indicator	Main results	Ref
Carbon metabolism flow, Iron metabolism flow	Considering direct and indirect emissions, Iron-Carbon metabolism nexus	RUE, PE, RE, LR, emission intensity, electricity consumption intensity	The CO_2_ emission are 2198.90 kg/t and 1582.40 kg/t in BOF and EAF, respectively.	This paper
Industrial metabolism, Mass and energy balance, Three-layer design	Hybrid carbon-hydrogen metallurgy manufacturing, carbon dioxide as targets	CO_2_ emission	The CO_2_ emission is 899.61 kg/t.	[26]
Mass and energy balance	Considering material, process and reaction conditions	Emission intensity, Energy efficiency	Energy efficiency is increased by 4.20%, CO_2_ emission reduced by 399.90 kg/t	[27]
Multi-objective optimization	Considering the effectiveness of the management strategies	Energy intensity, CO_2_ emission, cost	Energy intensity is 524.00 kgce/t, CO_2_ emission reduced by 125.03 kg/t	[28]

### 4.2 Evaluation of iron metabolism

**Fig 4:**
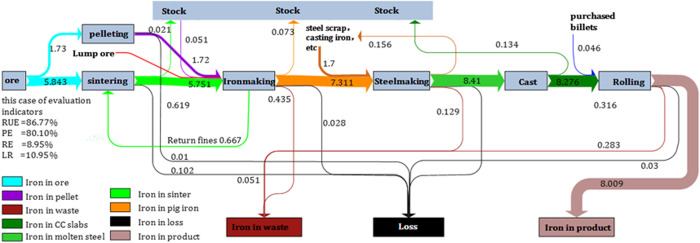
Iron metabolism flow (UnitMt).

To understand the iron flow more visually, the iron flow is represented based on iron metabolism paths by software Sankey. Fig 4 shows the metabolic network of iron, including sintering, pelletizing, ironmaking, steelmaking, casting and rolling process. Each process is marked with its input and output. The ore is the source of iron in the system. The input of sintering mainly includes 4 types of ore: domestic fine ore,imported fine ore, imported raw ore, return fines. The average grades of these ores are 0.65, 0.65, 0.62, 0.55, respectively. In addition, some recyclable iron-containing materials are also recycled, such as scale skin and muddy dust, removal dust and furnace dust. Constrained by actual production conditions, it is often necessary to pull additional material from stock in order to obtain higher metal yields. For example, partial sinter will be stored in inventory, while partial pellets will be transferred from inventory to be used in the ironmaking process. The iron stored in inventory accounts for 2.28% of the total input, classified as recyclable materials. After several processes, the final iron obtained is 8.01 Mt. The proposed evaluation indicators are showed in the Fig 4. From the perspective of the whole plant, the resource efficiency of iron is 86.77%, the product efficiency of iron is 80.10%, the reuse efficiency of iron is 8.95%, the loss rate of iron is 10.95%.

Since iron element came from raw ore in the iron and steel system, ferrous content of ore, and specific consumption of sinter will have more impact on the evaluation metrics. Under the same production equipment and production process conditions, inferior iron ore will lead to low efficiency in sintering, and then may lead to low tapping in ironmaking. Moreover, in order to remove impurities in the ore and ensure the output of pig iron, more raw materials need to be put in, thus increasing the burden of the process. On the contrary, higher ferrous content of ore means that the same quality of superior ore will obtain more iron products, less wastes and system loss (such as slags, iron ore slime & bottom ash/ fly ash). What’s more, as reported by Ref. [29], increasing ferrous content of ore in the ironmaking process can reduce the reducing agent ratio and accordingly diminish the consumption of coke. Additionally, the specific consumption of sinter can reflect the productivity of iron and steel process system. The higher the value, the lower the process level, which means the system needs to be improved. Low utilization efficiency of iron resources indicates that the process system is backward. As consequence, the indexes of LR and EM will rise. Moreover, as stated in Ref. [29], the increase of sinter specific consumption is accompanied by the increase of carbon dioxide intensity. Similarly, when the ore grade increased 1.00%, the sinter grade increased 0.98%, the limestone consumption reduced 14.30 kg/t crude steel, while dolomite increased 6.00 kg/t crude steel [30], coke ratio dropped by 2.00% [31]. This shows that ferrous content of ore has a greater influence on resource utilization efficiency, which is why high-grade ores and sinter must be used.

**Fig 5:**
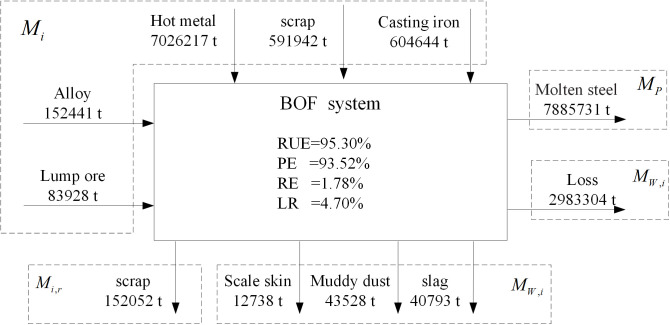
Metal input and output of BOF.

**Fig 6:**
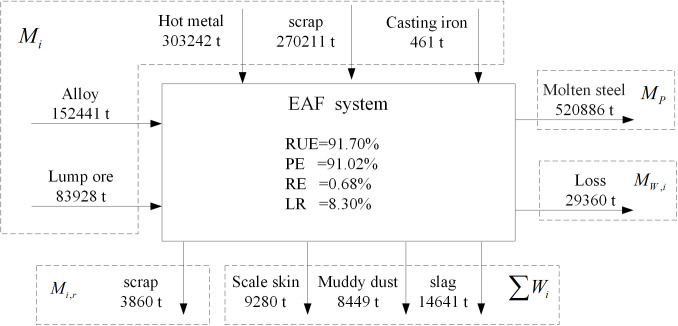
Metal input and output of EAF.

Figs 5 and 6 show the inputs and outputs of the two steelmaking systems. The resource utilization efficiency, production efficiency, reuse efficiency, system loss rate of iron for BOF steelmaking system are 95.30%, 93.52%, 1.78%, 4.70%, respectively. However, the resource utilization efficiency, production efficiency, reuse efficiency, system loss rate for EAF steelmaking system are 91.70%, 91.02%, 0.68%, 8.30%, respectively. The level of iron resource utilization in EAF steelmaking is much lower than that in BOF steelmaking system, however, the loss rate and recycling rate are much higher than that in BOF steelmaking system. To produce 1.00 t of product, the system loss is 37.70 kg/t, waste is 12.50 kg/t and scrap is 19.10 kg/t in the BOF system, while the system loss is 56.50 kg/t, waste is 34.70 kg/t and scrap is 7.40 kg/t in the EAF system. The EAF system has higher losses and waste, thus resulting in low resource utilization.

### 4.3 Evaluation of carbon metabolism

**Fig 7:**
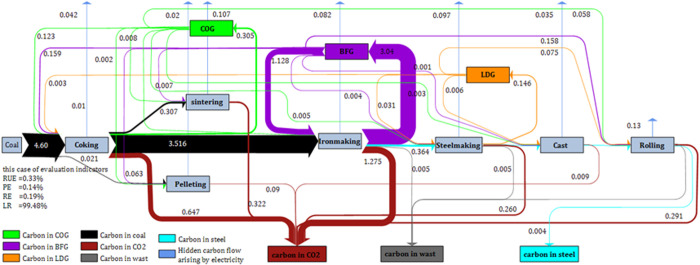
Carbon metabolism flow (UnitMt).

Fig 7 shows the diagram of carbon metabolic network and evaluation indicators. According to the results of carbon flow, carbon is mainly emitted into the atmosphere in the form of carbon dioxide. The resource efficiency of carbon is 0.33%, the product efficiency of carbon is 0.14%, the reuse efficiency of carbon is 0.19%, the loss rate of carbon is 99.48%. Dissimilar to iron, carbon mainly came from fuels mainly including cleaned coal, coke, injected coal powder, etc. This coking plant with self-production 3.37 Mt provides the energy needed for the whole iron and steel system. Coke ratio is 0.40 t/t crude steel in the case study, while the imported coke ratio is $0.05\times 10^{-1}$ t/t crude steel. As reported by Ref. [32], the increase of import coke ratio will lead to the decrease of carbon dioxide emission intensity. However, increasing the imported coke ratio, the energy intensity will increase [33]. This is because the energy consumption of imported cokes during production process are not considered. The energy consumption of imported coke must be considered [34], otherwise it will cause these companies appear to be more energy effectual than steel plants with own coking plants. In addition, the hidden carbon emissions cannot be ignored, especially the hidden emissions from electricity consumption.

**Fig 8:**
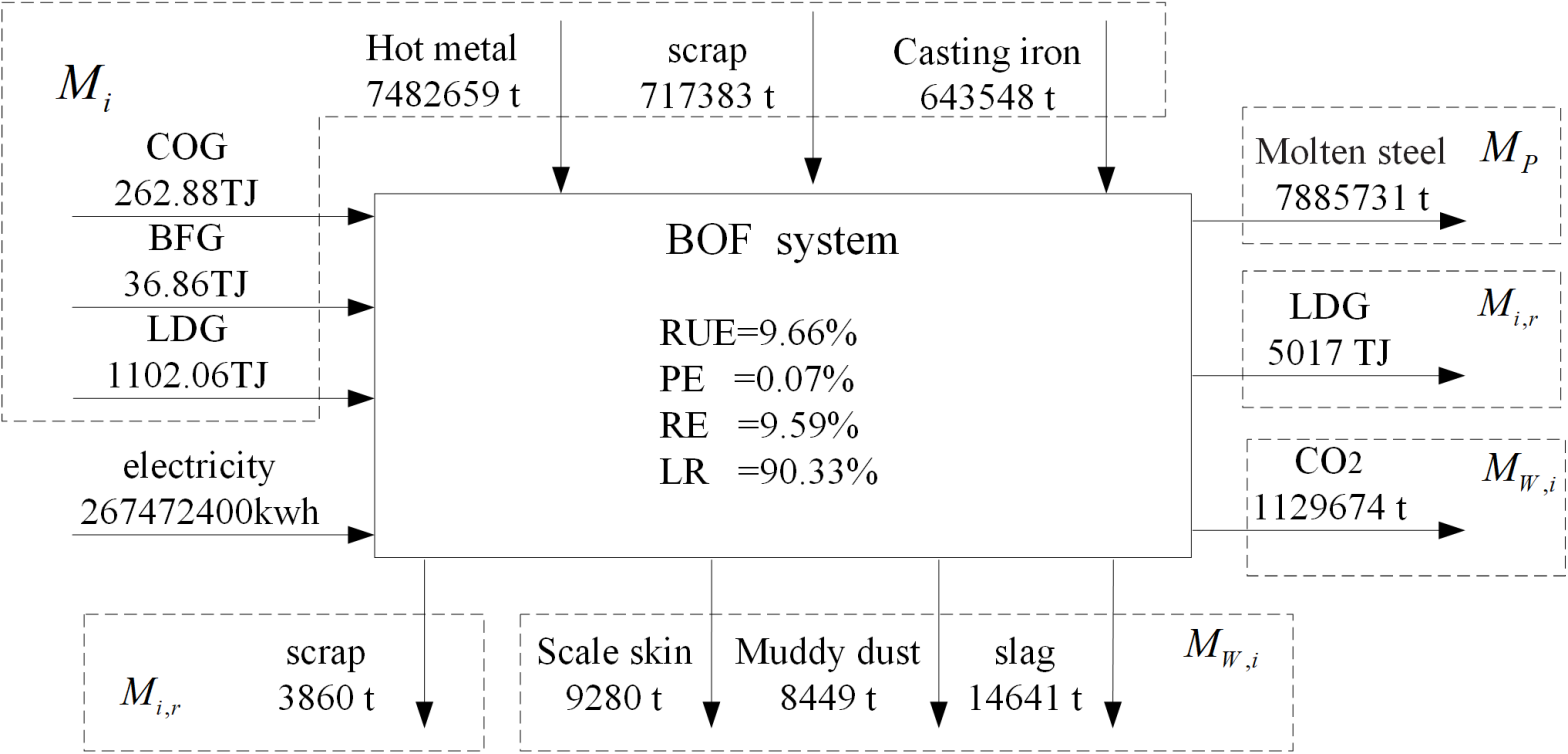
Main energy metabolism flow of BOF.

**Fig 9:**
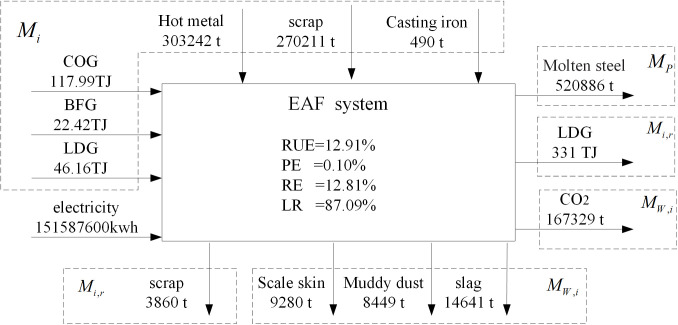
Main energy metabolism flow of EAF.

Similar to the iron metabolism, the carbon metabolism of the BOF and EAF steelmaking systems will be discussed and analyzed below. Carbon comes from fossil fuels in BF-BOF route. The coking process, as a fuel preparation system, provides a large amount of energy for the iron and steel system. However, EAF system uses a large amount of scrap steel as raw material and consumes huge electricity. The yield of the BF-BOF route accounts for 94.00% of the total yield, while the yield of the EAF route accounts for 6.00%. Moreover, the EAF needs to invest partial molten iron to reduce electricity consumption. Fig 8 shows the input and output of BOF steelmaking system. The evaluation indicators are as follows: the resource efficiency of carbon is 9.66%, the product efficiency of carbon is 0.07%, the reuse efficiency of carbon is 9.59%, the loss rate of carbon is 90.33%. Fig 9 shows the input and output of EAF steelmaking system. The evaluation indicators are as follows: the resource efficiency of carbon is 12.91%, the product efficiency of carbon is 0.10%, the reuse efficiency of carbon is 12.81%, the loss rate of carbon is 87.09%.

### 4.4 Iron-Carbon metabolism nexus

Iron metabolism and carbon metabolism are inextricably linked in iron and steel manufacturing systems. The purpose is to obtain higher metal yield with the least energy consumption. In order to understand the relationship between iron and carbon metabolism in BF-BOF route and EAF route, some comprehensive evaluation indicators are favored by companies and are proposed as follows.


$$\begin{array}{c} Emission\;intensity=carbon\;emission/molten\;steel\\ Electricity\;intensity=electricity/molten\;steel \end{array}$$ (7)

**Fig 10:**
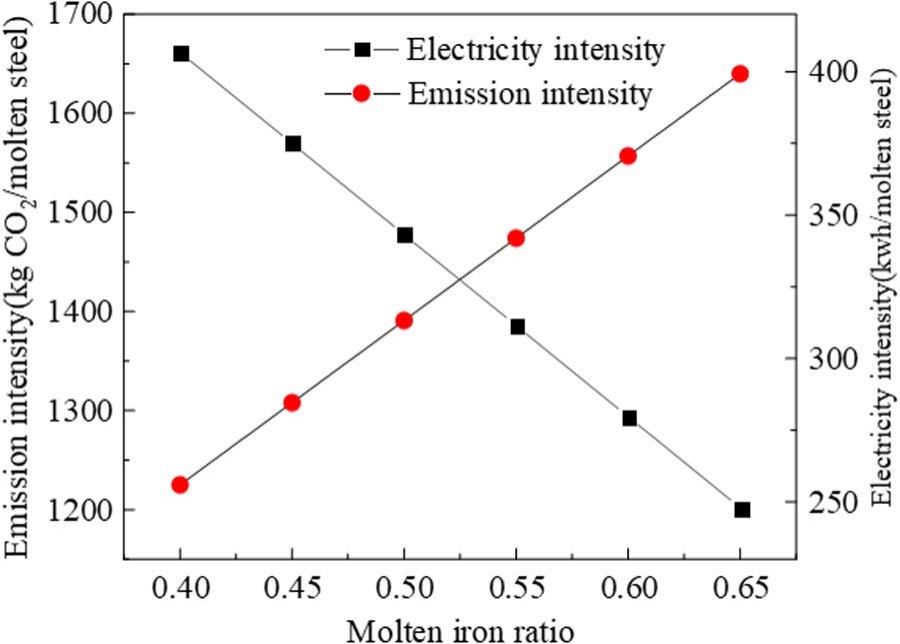
The impact of CO2 emission in EAF.

Fig 10 shows the relationship between molten iron ratio and emission intensity, electricity intensity. Considering the material and energy metabolism of the upstream process (ironmaking), the molten iron ratio is directly proportional to carbon emissions and inversely proportional to electricity consumption intensity. In order to reduce electricity consumption and scrap, the ratio of molten iron varies from 0.40 to 0.65 in EAF. The average emission intensity will decrease 83.00 $kgCO_{2}/moltensteel$ if the molten iron ratio decreases 0.05 in EAF, while electricity consumption intensity increases 32.00 kWh/moltensteel. The heat from molten iron will be replaced by electricity, resulting in the rise of electricity consumption intensity.

### 4.5 Potential ways to enhance metabolic efficiency

It is reported that the input of 1.00 t of scrap steel can reduce 1.60 t of iron ore, 0.35 t of standard coal and 1.60 t of CO_2_ emission. According to these conclusions, companies should vigorously develop EAF steelmaking. However, the fact is that China’s BF-BOF routes produces 88.40% of the total steel output, which is higher than the world average of 71.50%. For example, the steel plant in this paper is mainly based on BOF steelmaking system, and the annual output of BOF steelmaking system is 7.90 Mt, accounting for 94.00% of the total, while the annual output of EAF is 0.50 Mt, accounting for 6.00% of the total. Cost has been an important factor limiting the EAF steelmaking in China, such as scrap shortage, high scrap prices, huge power consumption, small capacity, etc. To produce 1.00 t of molten steel, the company’s EAF steelmaking consumes 333.00 kWh electricity. If the production is doubled(1.00 Mt), an additional $1.66\times 10^{8}$ kWh electricity is required. In the current tight situation of power supply, it is also a great challenge to have a stable power supply. The indirect carbon emissions from electricity consumption can not be ignored. Therefore, further study may be to optimize and minimize the carbon emissions.

## 5. Conclusions

Material and energy flow analysis of ISI is an effective way to explore energy saving and emission reduction. This paper proposed the SFA approach to analyze the industrial metabolism of iron and carbon in a state-owned and large-scaled steel enterprise in China. A widespread model is established and four evaluation indicators are proposed to appraise resource utilization efficiency. The results showed that the resource utilization efficiency of iron is 86.77%, and that of carbon is 0.33%. In EAF, the ratio of hot metal and the electricity consumption are the main factors affecting the carbon emission, so the comprehensive consideration of both can effectively reduce the carbon emission. These findings provide worthwhile reference for ISI to improve resource utilization and decrease emissions.

The contributions of this paper may provide valuable reference and ways for the iron and steel enterprises. Moreover, the proposed SFA and evaluation indicators can also be used in other iron and steel enterprises and even eco-industrial parks. The future work may be conducted in the following aspects: (1) The mechanism of iron and carbon from the perspective of industrial symbiosis can be studied in depth. (2) A multi-objective optimization model considering material efficiency, energy efficiency and carbon emissions can be established to achieve the optimal design of the ISI.

## Supporting information

S1 Data The original data of this paper. (XLSX)

## Abbreviations

BFG: Blast furnace gas
BF-BOF: blast furnace–basic oxygen furnace
COG: Coke Oven Gas
EAF: Electric arc furnace
IO: input-output
IPCC: Intergovernmental Panel on Climate Change
ISI: iron and steel industry
LDG: Linz—Donawitz Process Gas
LR: System loss rate
LCA: life cycle assessment
PE: Production efficiency
RUE: Resource utilization efficiency
RE: Reuse efficiency
SFA: Substance flow analysis
